# Review of major meat-borne zoonotic bacterial pathogens

**DOI:** 10.3389/fpubh.2022.1045599

**Published:** 2022-12-15

**Authors:** Sultan Ali, Abdullah F. Alsayeqh

**Affiliations:** ^1^Institute of Microbiology, Faculty of Veterinary Science, University of Agriculture, Faisalabad, Pakistan; ^2^Department of Veterinary Medicine, College of Agriculture and Veterinary Medicine, Qassim University, Buraidah, Saudi Arabia

**Keywords:** meat borne zoonoses, *Escherichia coli*, *Salmonella*, *Listeria*, *Brucella*, food intoxication, antimicrobial resistance, One Health approach

## Abstract

The importance of meat-borne pathogens to global disease transmission and food safety is significant for public health. These pathogens, which can cause a variety of diseases, include bacteria, viruses, fungi, and parasites. The consumption of pathogen-contaminated meat or meat products causes a variety of diseases, including gastrointestinal ailments. Humans are susceptible to several diseases caused by zoonotic bacterial pathogens transmitted through meat consumption, most of which damage the digestive system. These illnesses are widespread worldwide, with the majority of the burden borne by developing countries. Various production, processing, transportation, and food preparation stages can expose meat and meat products to bacterial infections and/or toxins. Worldwide, bacterial meat-borne diseases are caused by strains of *Escherichia coli, Salmonella, Listeria monocytogenes, Shigella, Campylobacter, Brucella, Mycobacterium bovis*, and toxins produced by *Staphylococcus aureus, Clostridium* species, and *Bacillus cereus*. Additionally, consuming contaminated meat or meat products with drug-resistant bacteria is a severe public health hazard. Controlling zoonotic bacterial pathogens demands intervention at the interface between humans, animals, and their environments. This review aimed to highlight the significance of meat-borne bacterial zoonotic pathogens while adhering to the One Health approach for creating efficient control measures.

## Introduction

**Figure 1:**
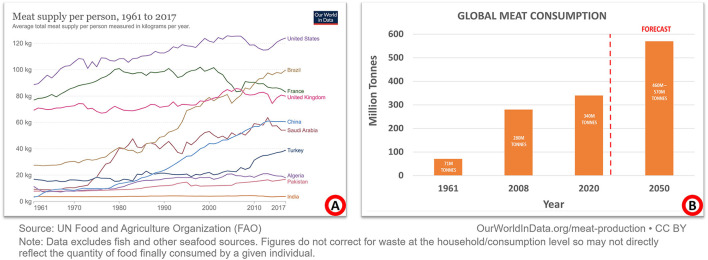
Global meat consumption and production since 1961. **(A)** Summarizes a select group of nations with annual meat consumption per person. Broadly speaking, the wealthier a country is, the more it consumes meat. Most people in low-income countries still consider meat a luxury item. **(B)** Summarizes global meat production/consumption. Compared to 1961, meat production is now four times as high. By 2050, it is predicted to rise to between 460 and 570 million metric tons.

Humans are considered omnivores because they have been eating meat for about 2.6 million years. Meat is a prominent source of protein in the average person's diet. Additionally, it has been shown that fortifying different lentils can ease the pressure on meat consumption (1). Since 1961, the amount of meat produced worldwide has more than quadrupled. Pork is the most consumed meat, although the poultry supply is expanding rapidly. Meat consumption varies widely among countries depending upon various factors, including the country's economy, culture, and more. Figure 1A depicts the amount of meat consumed by one individual in selected countries since 1961. It has been demonstrated that countries with a higher per capita income consume more meat than those with a lower per capita income. The world produces almost 340 million metric tons of meat a year, three times more than 50 years ago. Meat consumption is estimated to rise by 460–570 million metric tons by 2050, as described in Figure 1B. It is estimated that the global consumption of meat will exceed 328 million metric tons in 2021 (2). Pathogenic diseases, such as lumpy skin disease, are becoming increasingly common in key livestock countries, which poses a major threat to the global supply of meat and meat products (3).

Among the numerous microbes interacting with animals, some of these pathogens may become zoonotic and cause illness among humans, posing a threat to public health and the economy. Animal-derived food products, including milk, meat, and eggs, are considered essential components of human nutrition (4). However, food contaminated with pathogenic microbes may pose a serious threat to public health. These diseases can be as mild as self-limiting diarrhea or as fatal as cancerous conditions. It has been estimated that contaminated food is the source of illness for 1 out of 10 people (5). Food-borne infections are more common in children under five, who account for 40% of all cases, burdening the healthcare system and impeding a nation's socioeconomic development (6).

### Meat-borne diseases (MDBs)

Meat, red or white, from mammalian, avian, amphibian, aquatic, and reptilian species is consumed by humans as food. It is an excellent source of proteins, vitamins, and minerals and contains essential amino acids. Food products that are consumed raw are considered a direct source of food-borne infections. These include unpasteurized milk, raw eggs, undercooked meat, and uncooked shellfish (7). Depending on the animal's health and the hygienic conditions of the meat processing facilities, meat can be a source of many different pathogens. These pathogens can enter the food chain either by direct infection of animals or by contamination during meat handling, processing, and retailing due to poor personal hygiene and sanitary conditions (8).

Humans have learned from their experiences that eating the meat of diseased animals may lead to serious disease conditions. The importance of meat-borne diseases (MBDs) has been emphasized with the development of the meat industry (9). Meat-borne diseases can be of chemical or toxicological origin, zoonotic animal diseases, or environmental contaminations. Among these types, bacterial pathogens are the most important causative agents, whether as zoonotic diseases or environmental contaminations (10).

Several bacterial pathogens, including *E. coli, Salmonella, Campylobacter, Listeria monocytogenes, Yersinia enterocolitica, Brucella* species, *Mycobacterium bovis, Bacillus anthracis* or toxin-producing species like *Staphylococcus aureus, Clostridium* species, and *Bacillus cereus*, cause meat-borne disease either by infecting animals or contaminating meat during meat processing or handling (11). Animals, the environment, human handlers, and contaminated water used during processing can be the sources of these pathogens. Therefore, preventing pathogens in food animals and having strict policies for proper hygiene are mandatory for minimizing MBDs (12).

Identification of the correct source of infection is usually hard to establish because of the slow progression of signs and symptoms of MBDs. The causative agent of the disease can be identified by testing a sample of recently consumed food. However, it is hard to determine because the recently consumed food sample is not available for laboratory processing (13).

MBDs can be categorized into five types, i.e., infections, intoxications, allergies, metabolic food disorders, and idiosyncratic illnesses (14). Among these illnesses, infections and intoxications can affect almost every person. The remaining are comparatively less common. Consumption of contaminated meat can lead to various diseases that can be divided into GIT diseases and extra-GIT diseases. The bacterial pathogens causing GIT diseases include *E. coli, Salmonella, Campylobacter, Listeria*, and so on, while pathogens like *Brucella* and *Mycobacterium* can cause diseases other than GIT. Most cases of MBDs are due to gastrointestinal (GIT) problems, particularly small intestine issues that manifest as diarrhea.

### Diarrhea

Diarrhea is a GIT disease that can be caused by a variety of pathogens and their toxins. Most meat-borne infectious pathogens cause exhausting diseases such as severe diarrhea. The term “diarrhea” comes from the Greek phrase “diarrhea,” which means “to flow through.” An increased bowel movement, fluid contents, and fecal volume distinguish it. Ultimately, unabsorbed solutes increase intestinal movement, and abnormal intestinal structure results in diarrhea. Toxins from bacteria and the virulence factors of bacteria that multiply can also cause diarrhea (15). Intestinal microbiota plays a key role in fighting off infections and keeping the body healthy.

**Figure 2:**
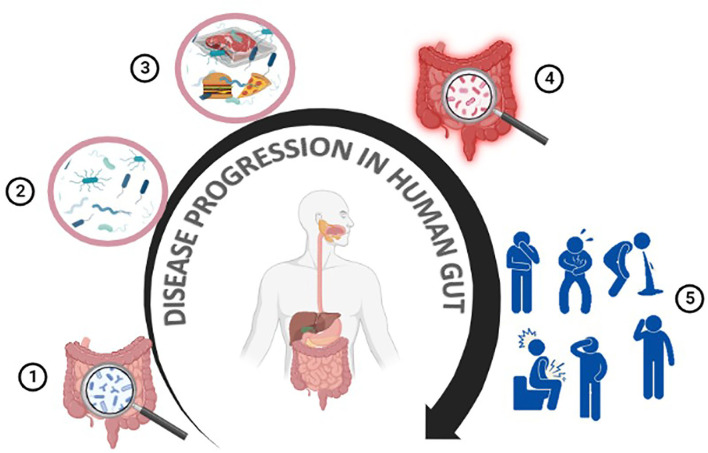
Meat-borne disease progression in humans. The human gut contains many beneficial/non-pathogenic microbes **(1,2)**. Meat or meat products can get contaminated at any point throughout the production, processing, or preparation steps **(3)**. Consuming contaminated food/meat or meat products can cause gastroenteritis, resulting in various health issues such as diarrhea, nausea, abdominal pain, and vomiting **(4,5)**.

In contrast, eating contaminated meat or meat products can cause severe inflammation of the gastrointestinal tract and a variety of related symptoms, such as nausea, vomiting, abdominal cramps, and diarrhea. A general mechanism of the food-borne GIT infection has been described in Figure 2. Bacterial infections can cause diarrhea through two distinct methods:

#### Toxigenic diarrhea

The physiological movement of the small intestine is disturbed by bacterial enterotoxins. By binding to epithelial cells, enterotoxins cause an increase in the secretion of electrolytes and a subsequent loss of water. This mechanism only results in secretory diarrhea because bacteria do not penetrate beyond epithelial tissues (16).

#### Invasive diarrhea

The severe dehydration that results from invasive diarrhea, caused by pathogen infiltration of the epithelial tissues lining the small or large intestine, is a leading cause of death among children worldwide. Penetration into deeper tissues and epithelial lining ulceration can induce dysentery (the appearance of blood in feces) (17).

### Meat-borne bacterial pathogens

**Figure 3:**
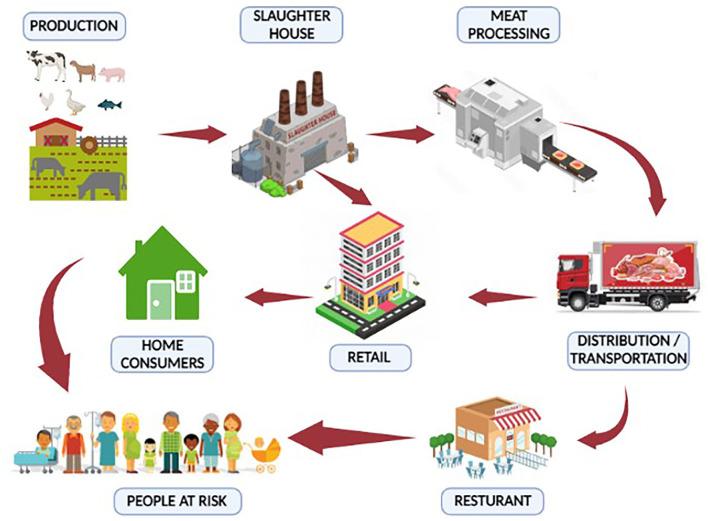
Meat supply chain (farm to fork). Meat or meat products can be contaminated with bacterial pathogens from “farm to fork”. The meat supply chain includes a production system for animals, a slaughterhouse, a meat processing unit, transportation, a retail market, and a restaurant. Stringent control measures should be adopted to minimize contamination and, hence, meat-borne diseases.

Mesophilic and psychotropic bacteria typically contaminate red and white meat. The majority of MBDs are zoonotic and transmitted to humans by direct or indirect contact. Meat or meat products go through several processes in the meat supply chain before they are consumed by the final consumers (18). Meat and meat products can be purchased by consumers at grocery stores or consumed at restaurants. Many community members can get sick by consuming contaminated meat and meat products (19). An overview of the meat supply chain from farms to consumers has been described in Figure 3 (20). Considering the One Health strategy for effective disease prevention, this article reviewed the most prevalent meat-borne bacteria and the diseases they cause.

#### Escherichia coli

*Escherichia coli* (*E. coli*) is a gram-negative, rod-shaped, non-spore-forming, facultatively anaerobe that was first isolated from a fecal sample in 1885 by Theodor Escherich. It is part of the normal microbiota of human and animal intestines. *E. coli* strains are classified based on H-antigen (flagella), O-antigen (somatic), and K-antigen (capsule). Currently, there are 174 somatic antigens, 80 capsular antigens, and 53 flagellar antigens that have been reported (22).

**Figure 4:**
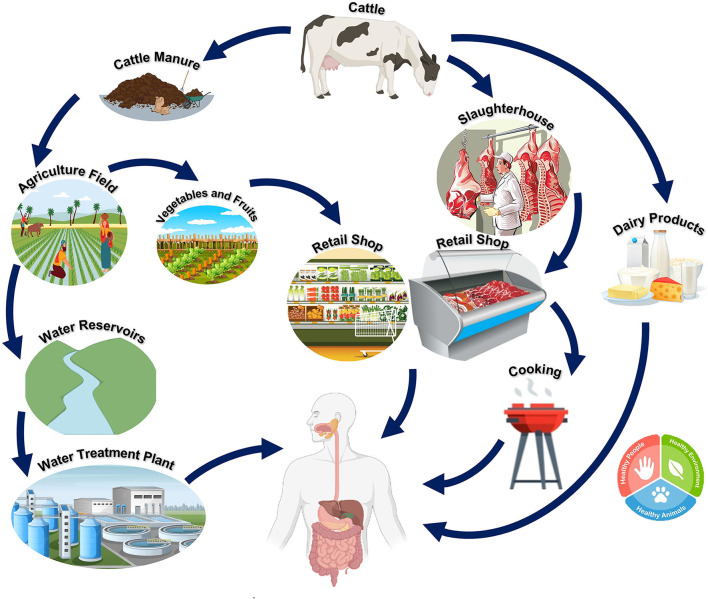
Reservoir and transmission of *E. coli* O157:H7. Cattle act as the main reservoir for O157:H7. The pathogen can be transmitted from cattle to humans directly (through the contamination of meat, meat, or dairy products from cattle) or indirectly [adapted from Vidovic and Korber (21)].

Pathogenic *E. coli* serotypes are usually associated with diarrhea or intestinal illnesses, but some strains of *E. coli* can also cause non-intestinal diseases. The primary source of this pathogen is the animal population, which is transferred to humans through animal products (23). Since the human food chain remains the primary transmission route for *E. coli* O157:H7 infection in humans, it is essential to stress the role that an intermediate habitat (i.e., a natural environment, in particular, the human food chain) would play in the fate of the clinical strains (24). These clinical strains of *E. coli* O157:H7 in hospitals are thought to be significantly influenced by the intermediate habitat. This zoonotic pathogen has been demonstrated to survive in its intermediate habitat, including the natural environment and the food matrix, after being excreted from its primary habitat (cattle). In this period, the intermediate habitat promotes the evolution of *E. coli* O157:H7 strains that can endure the harsh conditions of the human food chain and the natural environment, increasing pathogen fitness (21). Figure 4 depicts the spread and continuous presence of *E. coli* in the environment and in humans and animals.

Many *E. coli* strains, once thought to be harmless, have acquired pathogenic genes and have evolved into potentially harmful pathogens. Such pathogenic strains, listed in Table 1, can infect humans and animals. Intestinal epithelial cell lining may be damaged and compromised by these pathogenic strains, which in turn can lead to disease by disrupting ion pumps, altering cytoskeletal assembly, triggering cell death, and exacerbating fluid loss (26).

**Table 1:** Pathotypes of pathogenic *E. coli*, diseases, and important virulence toxins.

***E. coli* pathogenic strains**	**Disease**	**Important toxins**
**Intestinal pathogenic** ***E. coli***		
a. Enterohemorrhagic *E. coli* or Shiga toxin-producing *E. coli*	Diarrhea, hemorrhagic colitis, hemolytic uremic syndrome	Shiga-like toxins, enterohemolysin
b. Enterotoxigenic *E. coli*	Acute watery diarrhea	Heat stable toxin, Heat labile toxin, Shiga toxin
c. Enteroinvsive *E. coli* d. *Enteropathogenic E. coli* e. *Enteroaggregative E. coli*	Acute dysentery Acute and/or persistent diarrhea Persistent watery diarrhea	Enteroaggregative heat stable, plasmid-encoded toxin
f. Diffusely adherent *E. coli* g. Adherent invasive *E. coli*	Watery diarrhea in children Diarrhea, inflammatory bowel diseases	Enterotoxin Secreted autotransporter toxin
**Extraintestinal pathogenic** ***E. coli***		
a. Uropathogenic *E. coli* b. Sepsis-associated *E. coli*	Urinary tract diseases Sepsis	
c. Neonatal meningitis *E. coli*	Meningitis in newborns	Hemolysin (Hly)
d. Avian pathogenic *E. coli*	Colibacillosis in fowls	Invasion (Ibe)

These data were adapted from Kaper et al. (25).

Food animals have been the source of several disease outbreaks in developed and developing countries. *E. coli* infections are a common source of reported GIT illnesses, and many of these cases are traced back to eating contaminated meat (27). Among *E. coli* strains, Enterohemorrhagic *Escherichia coli* (EHEC) can cause life-threatening diseases due to hemolytic uremic syndrome and hemorrhagic colitis. The EHEC strain, O157:H7, can be differentiated from other *E. coli* strains by its inability to ferment sorbitol. EHEC is believed to be present in a wide variety of meat, poultry, lamb, pork, and raw milk products (28). Strict monitoring in light of the One Health approach can help reduce the spread. Additionally, prevention strategies such as cooking, practicing good hygiene, and avoiding potentially contaminated food might reduce the prevalence of this bacterium in the general population (29).

#### Salmonella

Salmonellosis, caused by the bacterial pathogen *Salmonella*, is one of the most common causes of mortality globally. *Salmonella* is a gram-negative, rod-shaped, non-lactose fermenting, non-spore-forming, and facultative anaerobe belonging to the family *Enterobacteriaceae*. The species of this genus do best between 35°C and 37°C (30). Many serotypes of *Salmonella* have been identified, although they can be divided into two species: *Salmonella enterica* and *Salmonella bongori* (31). Serotypes belonging to the *S. enterica* subspecies enterica account for 1,586 of the 2,659 serotypes (32).

*Salmonella* serotypes can be classified into three groups according to their ability to infect different hosts: host-restricted, host-adapted, and generalist. Host-restricted serovars can infect only a single type of host, causing typhoid-like disease. These include *S*. Pullorum and *S*. Gallinarum (poultry pathogens) and *S*. Typhi and *S*. Paratyphi (human pathogens) (33). Host-adapted serovars normally infect a single host but can also infect other host ranges. These include *S*. Dublin (a cattle pathogen) and *S*. Choleraesuis (a pig pathogen) (34). Generalist serovars can live in the gastrointestinal tracts of a wide variety of animals, rarely causing systemic infections (35). Non-typhoidal *Salmonella* (NTS) serovars are particularly widespread among these, infecting a wide variety of animal and human hosts. These NTS include *S*. Enteritidis and *S*. Typhimurium (36). The World Health Organization (WHO) has declared that NTS is a major threat to world health, particularly in low-income nations (37).

**Figure 5:**
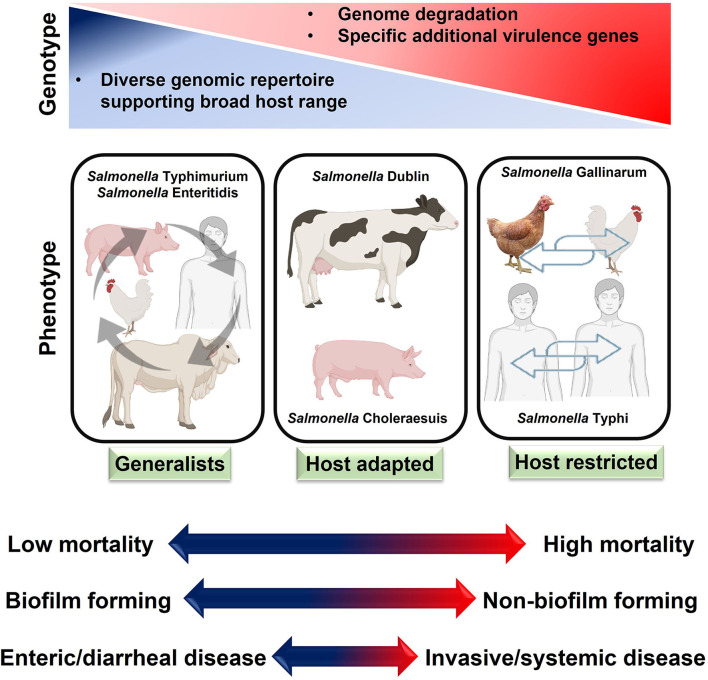
Host adaptation of salmonellae and disease characteristics. Generally, host-restricted salmonellae cause more severe systemic diseases than host-adapted and generalists *Salmonella*.

After interacting with microfold cells, typhoidal *Salmonella* spreads to lymphoid tissue and causes a systemic infection. Eventually, they disseminate throughout the body *via* the lymphatic or circulatory systems. In contrast, NTS serotypes are localized to the intestinal tract and provoke a strong immune response (38). Overall, host-restricted serovars of *Salmonella* are more pathogenic than host-adapted and generalist serovars. The phenotype, genotype, and systemic impacts of these serotypes have been summarized in Figure 5.

Antimicrobial medications can inhibit infections by disrupting their key function. Nonetheless, antimicrobial resistance (AMR) can develop when bacteria find ways to evade these medications (39). The increasing prevalence of antibiotic-resistant bacteria due to horizontal gene transfer is a major public health threat (40). Multiple drug-resistance serovars have developed resistance to three or more different classes of antimicrobials (41). Five percent of NTS isolates from human infections have been observed to be resistant to multiple drugs (42). A major threat to global public health is posed by multidrug-resistant *Salmonella* serovars, which are now again on the rise (43). Using alternatives to antibiotics in the feed may help slow the spread of AMR in animals (44).

**Figure 6:**
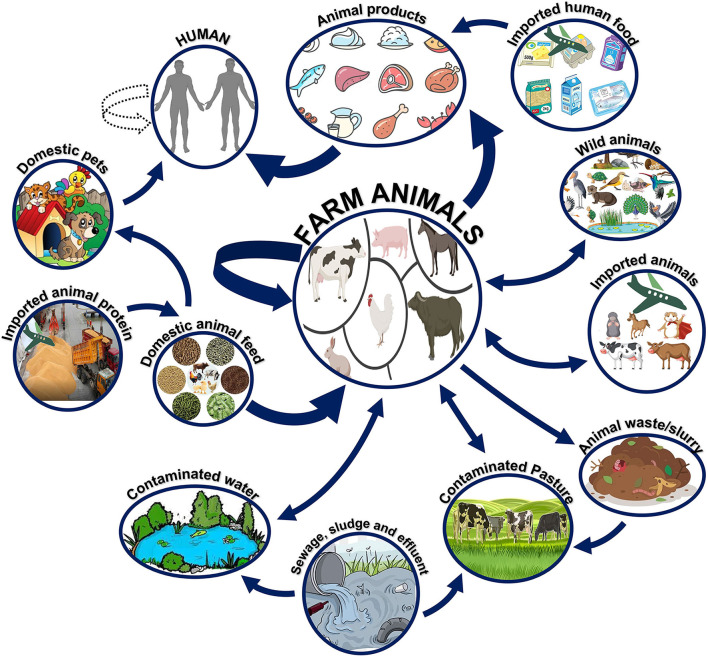
Reservoirs and transmission of non-typhoidal *Salmonella* (NTS). Farm animals serve as the main reservoirs for non-typhoidal *Salmonella*. The pathogen can survive in various animals or environments and eventually be transmitted to humans *via* consuming contaminated meat products or *via* direct contact with domestic animals.

Especially in the case of host-adapted serovars, most *Salmonella* infections in farm animals are acquired from animals of the same species. There are notable behavioral differences between *S*. Dublin and *S*. Typhimurium in adult cattle. The cases of *S*. Dublin clinical infection that resolve in the animal may become long-term carriers. It is possible that other herds are also infected but only show symptoms during times of high stress, especially during parturition (45). Experimental infection of calves by aerosol has also been reported, supporting the long-standing hypothesis that *Salmonella* may be transferred through the air (46). There have been numerous clinical cases in adult cattle because of grazing on recently contaminated pastures. Figure 6 summarizes how NTS can spread and persist at the point of contact between humans, animals, and their natural environments (47). Continuance of surveillance, early detection and management of sources, adequate hygienic measures, and execution of government rules and policies can help reduce the number of salmonellosis cases (48).

#### Campylobacter

*Campylobacter*, the bacteria responsible for campylobacteriosis, has a major impact on public health and the economy, especially in developing countries. It is gram-negative, curved, flagellated, microaerophile, catalase positive, and oxidase positive, and it can grow at an optimum temperature of 37–42°C. *C. jejuni* and *C. coli* are two of the most common species that cause disease in people. Researchers have linked these two species to both domestic and wild animals (49). *C. jejuni* is responsible for over 90% of all *Campylobacter* infections (50).

**Figure 7:**
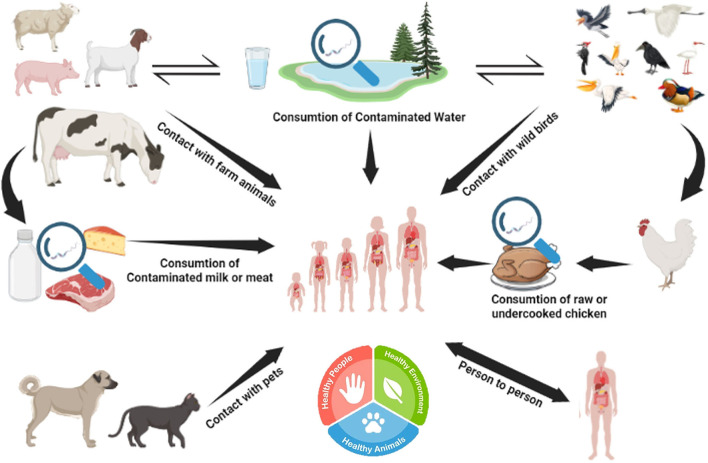
Reservoir and transmission of *Campylobacter*. *Campylobacter jejuni* can survive in various animals (poultry, cattle, and wild birds) and water reservoirs. Humans can acquire *C. jejuni* from consuming contaminated meat or meat products.

Poultry is a major natural reservoir of *C. jejuni*. Within poultry flocks, they spread through fecal-oral transmission (51). It is believed that only a low infectious dose (500–800) of *C. jejuni* is enough to induce GIT disease in humans (52). *C. jejuni* can contaminate water sources and thrive in domestic animals such as cattle and pork. Consumption of unpasteurized milk or undercooked meat can lead to GIT inflammation caused by *Campylobacter jejuni*, infecting the epithelial cells lining the intestine (53). The onset of the disease's symptoms might occur anywhere from 1 to 10 days after exposure. Patients with compromised immune systems are the only ones who are typically severely infected with *Campylobacter*-caused gastroenteritis (54). This pathogen can survive the hostile environment of the intestine due to various virulence factors, including motility, bile resistance factors, adhesion factors, and many different cytokines like cytolethal distending toxin (CTD) (55). Campylobacteriosis has a wide range of hosts and can be found in animal and environmental settings (see Figure 7 for an overview). Adaptation of proper hygienic practices during the handling of animals and animal products, such as fully cooked meat, can lower the risk of *Campylobacter* infections (56).

#### Shigella

Species of the genus *Shigella* are the pathogens that most often cause human dysentery. It is a gram-negative, rod-shaped, non-motile, non-lactose-fermenting, and facultative anaerobe pathogen. Table 2 shows how the pathogen has been classified into four categories based on the O antigen of the lipopolysaccharides. The genes for the lethal Shiga toxin are harbored in this pathogen (58). In immunocompromised patients, shigellosis can induce a severe form of the hemolytic uremic syndrome. *S. dysenteriae*, more than any other species, causes life-threatening shigellosis. It can be transmitted through the fecal-oral route due to poor sanitary practices. Only human beings are thought to harbor the pathogen (59). Using contaminated equipment, improper storage, and inadequate cooking can exacerbate the problem. The transmission of Shigellosis can be reduced by addressing the 5 “Fs,” i.e., food, fingers, feces, flies, and termites (60).

**Table 2:** *Shigella* serogroups and their characteristics.

**Species**	**Serogroup**	**Number of serotypes**	**Geographic location**	**Typical characteristics**
*Shigella dysenteriae*	A	15	Asia, Africa, Central America	Most severe dysentery with high mortality
*Shigella flexneri*	B	8	Common in developing states	Causes less severe dysentery than *S. dysenteiae*
*Shigella boydii*	C	19	Indian subcontinent mainly	Serologically different from *S. flexneri*
*Shigella sonnei*	D	1	Most common in developed stated	Causes mildest shigellosis

These data were adapted from Muthuirulandi Sethuvel et al. (57).

#### Listeria monocytogenes

Even though there are several species in the genus *Listeria*, most of them are harmless because they lack the virulence factors that make their hosts susceptible to infection. Due to its capacity to cause disease (listeriosis) in humans and animals, *Listeria monocytogenes* has been classified as a public health issue. It is a gram-negative, rod-shaped, facultative anaerobe, glucose-fermenting, and able to grow at a wide range of temperatures (0–45°C) (61).

At first, it was thought to be responsible for causing abortions and encephalitis in rabbits and pigs. After 50 years, it was also established as a human food-borne disease. It has been established that it can induce gastroenteritis in humans, abortions in women, and meningitis in immunocompromised people. *Listeria monocytogenes* cause a significant fatality rate, between 20 and 30% (62).

**Figure 8:**
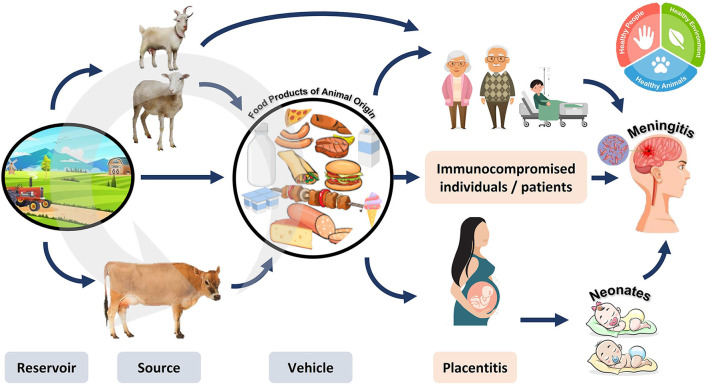
Reservoir, transmission, and diseases caused by *Listeria monocytogenes*. *Listeria monocytogenes* can circulate in animals and the environment. The pathogen can be transmitted to humans by consuming contaminated animal products. The resulting disease is more dangerous in pregnant women (causing inflammation of the placenta leading to abortion), neonates, and immunocompromised individuals (causing meningitis).

Several large-scale listeriosis outbreaks have been linked to the consumption of ready-to-eat (RTE) meat, seafood, and dairy products. Listeriosis can be contracted by eating contaminated food or encountering infected animals (63). Occasionally, it can be passed on to newborns. Its pathogenicity is based on its ability to proliferate within the cytoplasm upon phagocytosis. The survival of pathogens within phagocytes is ensured by the actions of listeriolysin O (LLO) and phospholipase (PlcA) (64). The pathogen can evade the humoral cell immune response by moving from cell to cell. Even in healthy people, listeriosis can cause a wide spectrum of symptoms, from mild diarrhea to deadly meningitis in those with compromised immune systems, as summarized in Figure 8 (65).

#### Yersinia enterocolitica

*Yersinia enterocolitica*, the causative agent of food-borne/meat-borne enteritis, along with *Yersinia pestis*, the causative agent of plague, and *Yersinia pseudotuberculosis*, belong to the genus *Yersinia*. Yersiniosis is caused by *Y. enterocolitica* and is often a self-limiting disease, but it can cause severe consequences post-infection in immunocompromised individuals (66).

After infecting small intestinal M cells, *Y. enterocolitica* travels through the body's lymphatic system. Antiphagocytic virulence factors are used to evade the host's immunological response. *Y. entercolitica* is capable of causing mild to moderate severity disease depending on host age, immune system condition, and environmental factors; however, it is rarely fatal (67).

**Figure 9:**
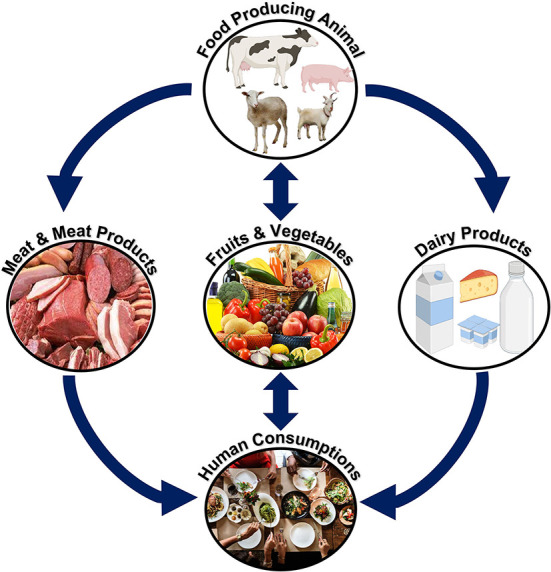
Transmission of *Y. enterocolitica* in humans. *Y. enterocolitica* can be transmitted to humans from animals through the consumption of contaminated food/meat or meat/dairy products.

*Yersinia* strains that are pathogenic to humans are primarily found in animal reservoirs, but they hardly ever cause illness in animals. Consumption of contaminated food, meat, and water can cause human disease. In addition, the waste products of food animals can potentially spread disease to humans when they contaminate fruit and vegetables (68). Figure 9 provides a concise summary of these animal food sources of the pathogen (69).

#### Brucella abortus

*Brucella* species are coccobacilli, which are Gram-negative, non-motile, aerobic, and non-spore-forming bacteria. These facultative intracellular bacteria exacerbate severe illnesses in animals and humans (70). Some of the most notable species in this genus and the animals that host them are *B. melitensis* (sheep and goats), *B. ovis* (sheep), *B. suis* (pigs), and *B. abortus* (cattle). Only *B. melitensis, B. suis*, and *B. abortus* can cause human brucellosis. *Brucella melitensis* is the most common species that causes brucellosis in humans, partly because of difficulties in immunizing free-ranging goats and sheep (71).

Brucellosis is common in many parts of the world, including Asia, South America, the Middle East, and Africa. Every year, more than half a million human cases are reported worldwide (72). Additionally, this is probably an underestimate because brucellosis cases are underreported and frequently misdiagnosed due to the lack of specific symptoms, the possible lack of awareness among physicians, and the limited diagnostic capabilities of laboratories (73). People having direct contact with animals, animal products, or people working in a laboratory with animals are at greater risk of exposure to brucellosis (74, 75).

**Figure 10:**
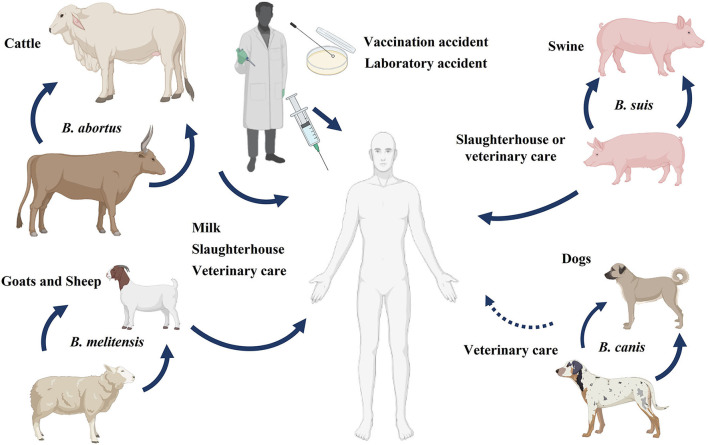
Reservoir and transmission of *Brucella*. Brucellosis in humans can be transmitted from different animals like cattle, sheep, goats, swine, and rarely from dogs. The sources of disease transmission include consumption of unpasteurized milk and milk products, direct association with animals or animal products, and accidental laboratory exposure.

Farmers, veterinarians, butchers, laboratory workers, milkers, and inseminators all risk contracting an infection at work due to their proximity to animals (76). Most field veterinary assistants, abattoir workers, and people working in many rural pastoral settings routinely handle aborted materials or attend to cases of retained placenta or dystocia without wearing protective gear. As a result, if the disease is present in domestic animals, it may also pose a significant threat to rural communities and animal health workers. Moreover, laboratory workers can accidentally acquire brucellosis while handling bacterial samples or cultures, as summarized in Figure 10 (77).

This pathogen only needs 10–100 cells to infect a human, but it can cause a debilitating, long-term illness (78). This pathogen can infect and replicate within a wide variety of host cells. Due to the fluctuating nature of the fever, this condition is also known as “undulant fever” when it occurs in humans. The disease is characterized by flu-like symptoms, such as a high temperature, a sore throat, a cough, a headache, a sore body, joint pain, sweating, fatigue, and anorexia (79). Adaptation of proper sanitary measures, safe handling of animals and animal products, and consumption of pasteurized milk can reduce the risk of brucellosis. It is imperative to re-establish a strict animal and human surveillance program by applying One Health principles (80).

#### Mycobacterium bovis

**Figure 11:**
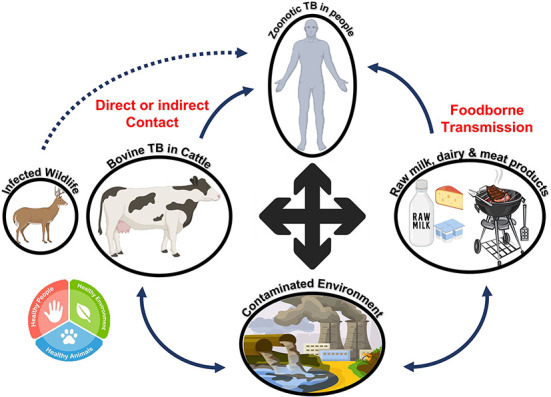
Reservoir and transmission of zoonotic tuberculosis. Bovine tuberculosis can be transmitted to humans directly or through contaminated dairy or meat products. The pathogen can survive in the environment and then enter the food chain, resulting in tuberculosis in humans after transmission.

Since ancient times, people have known that tuberculosis can be transmitted between humans and animals. The bacterium *Mycobacterium tuberculosis* is responsible for most human tuberculosis cases. On the contrary, *M. bovis* is responsible for bovine tuberculosis. However, *M. bovis* can also infect humans because the disease can be transmitted from animals (81). The capacity of the *Mycobacterium* to replicate in a diverse range of hosts exacerbates the situation (82). To resist immunological clearance by the host, mycobacteria significantly modify the innate defense systems employed by the host immune system (83). Zoonotic tuberculosis can be transmitted from animals to humans through direct contact with infected animals, exposure to contaminated environments, and consumption of contaminated dairy and meat products (84). People who lack an efficient immune system have a greater chance of contracting the infection. As shown in Figure 11, the One Health approach should be used to reduce the number of infectious agents encountered in people, animals, and their environments (85).

### Meat-borne intoxication

Toxins produced by pathogenic bacteria naturally found in or transmitted to meat or meat products can cause meat-borne intoxication (86). Some bacteria can survive high temperatures or enter food even after it has been cooked or pasteurized. During the food-borne multiplication of these pathogens, they can generate neurotoxins and enterotoxins. Bacterial pathogens like *Staphylococcus aureus, Bacillus cereus*, and *Clostridium perfringens* are examples of this group (87). *S. aureus* is the most common cause of food poisoning from contaminated meat.

#### Staphylococcus aureus

The food intoxication caused by a member of the *Staphylococcus* family is commonly termed staphylococcal food poisoning (SPF). There are more than 50 recognized species and subspecies of *Staphylococcus* (88). Staphylococcal species are classified as either “coagulase positive” or “coagulase negative” based on whether or not they produce the coagulase enzyme. The coagulase enzyme acts as a virulence factor by converting prothrombin into staphylothrombin and plasma fibrinogen into fibrin, helping bacteria evade the immune response (89).

*S. aureus* produces a diverse array of virulence factors and toxins. Among these factors, Staphylococcus enterotoxins (SEs) are responsible for food poisoning commonly produced by coagulase-positive *S. aureus*. Among the 23 different SEs, some cause pyrogenic disorders, enteritis, and food poisoning. These plasmid-mediated toxin genes can easily be transmitted horizontally to non-virulent strains, altering them into virulent strains (90). Staphylococcal toxins (A–E) are called classic enterotoxins except for SE–F due to their structural similarity with toxic shock syndrome toxins (91). The two most prevalent food poisonings are associated with SEA and SEB. The following Table 3 describes several different SEs along with their associated pathologies:

**Table 3:** Superantigens produced by *S. aureus*, along with associated pathology and genes.

**Superantigens**	**Associated pathology**	**Associated gene**
Enterotoxin A	Enteritis, food poisoning	*Sea*
Enterotoxin B	Enteritis, food poisoning	*Seb*
Enterotoxin C	Enteritis, food poisoning	*Sec*
Enterotoxin D	Enteritis, food poisoning	*Sed*
Enterotoxin E	Food poisoning	*See*
Enterotoxin G	Food poisoning	*Seg*
Enterotoxin H	Food poisoning	*She*
Enterotoxin I	Food poisoning	*Sei*
Enterotoxin F / TSST_−1_	Toxic shock syndrome	*Tst*

These data were adapted from Fisher et al. (92).

**Figure 12:**
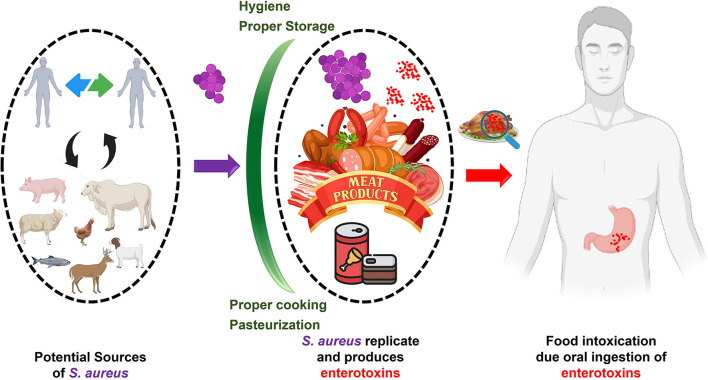
Staphylococcal food poisoning (SFP)/intoxication. *Staphylococcus aureus* **(Violet)** can enter food from various sources. Improper storage may lead to the replication of *S. aureus* and the production of enterotoxins **(Red)**. The consumption of enterotoxin-contaminated food (intoxication) may lead to gastroenteritis. Possible intoxication can be inhibited by proper cooking, pasteurization, and storage **(Green barriers)**.

Consumption of SEs-contaminated meat and meat products causes food-borne staphylococcal intoxications (93). Depending on the sensitivity and immune condition of the affected person, as little as 0.1 μg of SEA toxin is enough to cause intoxication (94). Implementing proper hygienic practices during food processing and handling can reduce the risk of food-borne intoxication. To help reduce the risk of staphylococcal food poisoning, Figure 12 [adapted from (95)] summarizes its likely causes, transmission pathways, consequences, and preventative measures.

### One Health and food safety

Global increases in the production and consumption of animal products are inevitable, given proportional gains in wealth and technological advancements in livestock and poultry production. Concerns have been raised about the capacity of existing animal and public health infrastructures to support the Livestock Revolution's rapid expansion of animal agricultural production systems in developing countries (96).

The idea of “One Health” has been around for a while, but it has recently reemerged as a crucial framework for comprehending and responding to the health problems of the modern era. The term “One Health” refers to a multi-pronged approach to improving the wellbeing of humans, animals, and the environment. Our world is dynamic, intricate, and increasingly interconnected. Health in all three groups is now intricately intertwined owing to the unique dynamic formed by the confluence of humans, animals, and the environment (97).

The relationship between humans and animals, especially animal products, is evolving because of these shifting dynamics. Human interaction with animals is booming, spreading, and having greater and greater consequences. As a result, we now have an impressive global food system that is both an agricultural and business achievement and a tremendous challenge that endangers the health of humans, animals, and the environment (98). While improvements in preventing and reducing food-borne diseases and other forms of food contamination have been made, they have been inconsistent, short-lived, and extremely challenging to sustain. New dimensions of cooperation, insight, and imagination will undoubtedly be required to guarantee a safe food supply (99). One Health is an attractive and contemporary approach because it takes a more proactive and integrated approach to finding and implementing solutions. It is imperative to create a new framework for preventing food-borne diseases rather than only reacting to them when they occur (100).

Embedded in this complex system is the potential role of food as a vehicle for disease transmission; food safety has grown in significance and become a vital public health commitment. This is a reminder that even though bacterial contamination of food is a major problem, other pathogens such as viruses, parasites, poisons, prions, chemicals, metals, and allergies can spread through consumption. Meat is gaining relevance as a vector for food-borne diseases; however, animal reservoirs are frequently the source of these infections. The concept of “One Health” provides the appropriate framework to examine this connection and, more significantly, gain a novel understanding that can help us modify our existing interventions and preventive measures (101).

For example, regarding the dangers of consuming certain types of food, we tend to concentrate on the potential effects on human health, even if the most efficient methods of preventing the spread of certain illnesses lie in the control measures taken. Microbes do not discriminate between species; they only want to survive and multiply. Our bias and artificial divide between animal and public health is a barrier to recognizing One Health. As a subset of this wider trend, the security of our food supply is becoming increasingly threatened by both deliberate and accidental contaminations and shifting microbial ecosystems (102).

The prevention and management of zoonotic diseases spread through meat can be enhanced by collaboration and cooperation under One Health concept. Because meat contamination can occur at various steps of the production and processing of meat, preventing meat-borne zoonotic diseases requires a comprehensive approach spanning from the point of production (at the farm) to the point of consumption (on the table). For example, cattle can be infected with *E. coli* from feed or the environment (at the farm), or beef can be contaminated with intestinal contents or the environment during slaughtering and processing (103).

## Conclusion

Meat-borne infections are a serious threat to public health in both developed and developing countries due to the widespread consumption of contaminated meat and meat products. Proper hygienic standards and stringent production, processing, and handling precautions are required to limit the risk of meat-borne diseases due to the transmission of pathogens from animals to people. Animal disease control efforts that fail to address the underlying causes of the disease may increase the likelihood of AMR in bacterial pathogens. Because AMR is rising and poses a risk to public health, it is important to use antibiotics judiciously in animal production and treatment. Vaccination of food handlers and animals and the application of the Codex Alimentarius Commission should be followed to decrease the prevalence of food-borne pathogens and infections. One Health approach approved by WHO, FAO, and OIE can present solutions to reduce food-borne diseases concerning public health. In conclusion, political will is required to ensure that the agreed policies are implemented.

## Author contributions

AA and SA co-wrote the review and approved the final draft. Both authors contributed to the article and approved the submitted version.

## References

[1] Aimon SS Liaquat A Aamir SH Qadeer T Ashraf S Ali R. Blend of flour with *Vigna radiata* and *Vigna mungo* used in muffins in order to increase nutritional properties. Agrobiol Rec. (2021) 3:29–35. 10.47278/journal.abr/2020.012

[2] Birgani RA Kianirad A Shab-Bidar S Djazayeri A Pouraram H Takian A. Climate change and food price: a systematic review and meta-analysis of observational studies, 1990-2021. Am J Clim Change. (2022) 11:103–32. 10.4236/ajcc.2022.112006

[3] Khan A Du XX Hussain R Kwon OD. Lumpy skin disease: a threat to the livestock industry - a review. Agrobiol Rec. (2022) 9:22–36. 10.47278/journal.abr/2022.015

[4] Mughini-Gras L Kooh P Augustin JC David J Fravalo P Guillier L. Source attribution of food-borne diseases: potentialities, hurdles, and future expectations. Front Microbiol. (2018) 9:1983. 10.3389/fmicb.2018.01983 30233509 PMC6129602

[5] Dhama K Rajagunalan S Chakraborty S Verma AK Kumar A Tiwari R. Food-borne pathogens of animal origin-diagnosis, prevention, control and their zoonotic significance: a review. Pak J Biol Sci. (2013) 16:1076–85. 10.3923/pjbs.2013.1076.1085 24506006

[6] Heredia N García S. Animals as sources of food-borne pathogens: a review. Anim Nutr. (2018) 4:250–5. 10.1016/j.aninu.2018.04.006 30175252 PMC6116329

[7] Roselli M Natella F Zinno P Guantario B Canali R Schifano E. Colonization ability and impact on human gut microbiota of food-borne microbes from traditional or probiotic-added fermented foods: a systematic review. Front Nutr. (2021) 8:689084. 10.3389/fnut.2021.689084 34395494 PMC8360115

[8] Ijaz M Yar MK Badar IH Ali S Islam MS Jaspal MH. Meat production and supply chain under COVID-19 scenario: current trends and future prospects. Front Vet Sci. (2021) 8:660736. 10.3389/fvets.2021.660736 34026895 PMC8137951

[9] Nkosi D Bekker J Hoffman L. Impact of communal cattle farming practices on meat safety in central bushbuckridge, South Africa. Int J Vet Sc. (2020) 9:90–6. Available online at: https://www.ijvets.com/pdf-files/Volume-9-no-1-2020/90-96.pdf

[10] Wang F Zhang W Niu D. Foodborne enterobacteriaceae of animal origin. Front Cell Infect Microbiol. (2021) 11:772359. 10.3389/fcimb.2021.772359 34650935 PMC8505891

[11] Bintsis T. Food-borne pathogens. AIMS Microbiol. (2017) 3:529. 10.3934/microbiol.2017.3.529 31294175 PMC6604998

[12] Lyu N Feng Y Pan Y Huang H Liu Y Xue C. Genomic characterization of *Salmonella enterica* isolates from retail meat in Beijing, China. Front Microbiol. (2021) 12:636332. 10.3389/fmicb.2021.636332 33897640 PMC8058101

[13] Elbediwi M Shi D Biswas S Xu X Yue M. Changing patterns of *Salmonella enterica* serovar Rissen from humans, food animals, and animal-derived foods in China, 1995–2019. Front Microbiol. (2021) 12:702909. 10.3389/fmicb.2021.702909 34394048 PMC8358327

[14] Abebe E Gugsa G Ahmed M. Review on major food-borne zoonotic bacterial pathogens. J Trop Med. (2020) 2020:4674235. 10.1155/2020/4674235 32684938 PMC7341400

[15] Camino Feltes MM Arisseto-Bragotto AP Block JM. Food quality, food-borne diseases, and food safety in the Brazilian food industry. Food Qual Saf. (2017) 1:13–27. 10.1093/fqs/fyx003

[16] Tortora et al. Microbiology: An Introduction. 13th ed. London, UK: Pearson Education Limited (2021).

[17] Chess B. Talaro's Foundations in Microbiology. 11th ed. New York, NY: McGraw Hill (2020).

[18] Nespolo NM. The behavior of consumers and producers of food of animal origin and their impacts in one health. Front Vet Sci. (2021) 8:607. 10.3389/fvets.2021.641634 34195242 PMC8236503

[19] El Jalil MH Khamar M Maaninou S Dahha M Zinedine A Ameur N. Antibiotic resistance of *Escherichia coli* strains isolated from broiler meat in Morocco. Int J Vet Sci. (2020) 9:305–8. 10.37422/IJVS/20.015

[20] Collineau L Chapman B Bao X Sivapathasundaram B Carson CA Fazil A. A farm-to-fork quantitative risk assessment model for *Salmonella* Heidelberg resistant to third-generation cephalosporins in broiler chickens in Canada. Int J Food Microbiol. (2020) 330:108559. 10.1016/j.ijfoodmicro.2020.108559 32599476

[21] Vidovic S Korber DR. *Escherichia coli* O157: Insights into the adaptive stress physiology and the influence of stressors on epidemiology and ecology of this human pathogen. Crit Rev Microbiol. (2016) 42:83–93. 10.3109/1040841X.2014.889654 24601836

[22] Tuo X Wang S Cui D Wang F Liu Y Wang H. Antibiotic resistance profiles and virulence markers of Escherichia coli strains isolated from diarrheal lambs in Gansu and Qinghai, China. Pak Vet J. (2020) 40:123–6. 10.29261/pakvetj/2019.102

[23] Moxley RA Bargar TW Kachman SD Baker DR Francis DH. Intimate attachment of *Escherichia coli* O157: H7 to urinary bladder epithelium in the gnotobiotic piglet model. Microorganisms. (2020) 8:263. 10.3390/microorganisms8020263 32075320 PMC7074727

[24] Ramos TDM Jay-Russell MT Millner PD Baron JN Stover J Pagliari P. Survival and persistence of food-borne pathogens in manure-amended soils and prevalence on fresh produce in certified organic farms: a multi-regional baseline analysis. Front Sust Food Syst. (2021) 5:674767. 10.3389/fsufs.2021.674767

[25] Kaper J Nataro J Mobley H. Pathogenic *Escherichia coli*. Nat Rev Microbiol. (2004) 2:123–40. 10.1038/nrmicro818 15040260

[26] Wang X Zhang H Bi L Xi H Wang Z Ji Y. Isolation, characterization and genome analysis of a novel virulent Escherichia coli bacteriophage vb_ecom_011d4. Agrobiol Rec. (2021) 6:27–35. 10.47278/journal.abr/2021.014

[27] Abd El-fatah SS Saad AS. Study on dispersal of *Escherichia coli* and *Salmonella enterica* in retail beef and chicken meat. Int J Vet Sci. (2020) 9:309–12. 10.37422/IJVS/20.023

[28] Tahir A Khan MA Bibi K Bibi S Rauf F Ayaz F. Prevalence of colibacillosis in young broiler chicks and antibiogram of *Escherichia coli* in different areas of Hazara Region. Adv Life Sci. (2021) 8:238–40. Available online at: https://www.als-journal.com/835-21/

[29] Byrne L Kaindama L Bentley M Jenkins C Aird H Oliver I. Investigation into a national outbreak of STEC O157: H7 associated with frozen beef burgers, UK (2017). Epidemiol Infect. (2020) 148:e215. 10.1017/S0950268820001582 32669142 PMC7522850

[30] El-Aziz A Norhan K Tartor YH Gharieb R Erfan AM Khalifa E. Extensive drug-resistant Salmonella enterica isolated from poultry and humans: prevalence and molecular determinants behind the co-resistance to ciprofloxacin and tigecycline. Front Microbiol. (2021) 12:738784. 10.3389/fmicb.2021.738784 34899627 PMC8660588

[31] Guerrini A Mescolini G Roncada P Tosi G Raffini E Frasnelli M. Seroprevalence and microbiological monitoring in eggs for *Salmonella enterica* serovar Enteritidis and *Salmonella enterica* serovar Typhimurium in ornamental chicken flocks in Italy. Pak Vet J. (2021) 41:39–44. 10.29261/pakvetj/2020.095

[32] Issenhuth-Jeanjean S Roggentin P Mikoleit M Guibourdenche M De Pinna E Nair S. Supplement 2008–2010 (no. 48) to the white–Kauffmann–Le minor scheme. Res Microbiol. (2014) 165:526–30. 10.1016/j.resmic.2014.07.004 25049166

[33] Xin S Zhu H Tao C Zhang B Yao L Zhang Y. Rapid detection and differentiating of the predominant *Salmonella* serovars in chicken farm by TaqMan multiplex real-time PCR assay. Front Cell Infect Microbiol. (2021) 11:759965. 10.3389/fcimb.2021.759965 34660351 PMC8512842

[34] Carroll LM Pierneef R Mathole M Matle I. Genomic characterization of endemic and ecdemic non-typhoidal *Salmonella enterica* lineages circulating among animals and animal products in South Africa. Front Microbiol. (2021) 12:748611. 10.3389/fmicb.2021.748611 34671335 PMC8521152

[35] Nawaz Z Aslam B Zahoor MA Siddique AB Rafique A Aslam R. Frequency of extended spectrum beta lactamase producing *Escherichia coli* in fresh and frozen meat. Pak Vet J. (2021) 41:102–6. 10.29261/pakvetj/2020.059

[36] Hassan M Ali A Ahmad A Saleemi MK Wajid M Sarwar Y. Purification and antigenic detection of lipopolysaccharides of *Salmonella enterica* Serovar Typhimurium isolate from Faisalabad, Pakistan. Pak Vet J. (2021) 41:434–8. 10.29261/pakvetj/2021.046

[37] Sadiq S Mansur-ud-Din Ahmad MC Akbar H Mushtaq MH Shehzad F Hassan S. Molecular epidemiology of zoonotic *Salmonella* Enteritidis isolated from poultry and human sources by Multi Locus Sequence Typing. Pak Vet J. (2020) 40:264–8. 10.29261/pakvetj/2020.103

[38] Schultz BM Melo-Gonzalez F Salazar GA Porto BN Riedel CA Kalergis AM. New insights on the early interaction between typhoid and non-typhoid *Salmonella* serovars and the host cells. Front Microbiol. (2021) 12:647044. 10.3389/fmicb.2021.647044 34276584 PMC8282409

[39] Pinheiro REE Chaves TP Melo ES Ali S Ali SW Umer M. Modulatory-antibiotic activity of the essential oil from *Eucalyptus citriodora* against MDR bacterial strains. Cell Mol Biol. (2020) 66:60–4. 10.14715/cmb/2020.66.4.10 32583772

[40] Do KH Byun JW Lee WK. Antimicrobial resistance, adhesin and toxin genes of porcine pathogenic *Escherichia coli* following the ban on antibiotics as the growth promoters in feed. Pak Vet J. (2021) 41:519–23. 10.29261/pakvetj/2021.067a

[41] Mehmood K Bilal RM Zhang H. Study on the genotypic and phenotypic resistance of tetracycline antibiotic in *Escherichia coli* strains isolated from free ranging chickens of Anhui Province, China. Agrobiol Rec. (2020) 2:63–8. 10.47278/journal.abr/2020.014

[42] Bahramianfard H Derakhshandeh A Naziri Z Khaltabadi Farahani R. Prevalence, virulence factor and antimicrobial resistance analysis of *Salmonella* Enteritidis from poultry and egg samples in Iran. BMC Vet Res. (2021) 17:196. 10.1186/s12917-021-02900-2 34030671 PMC8142639

[43] Sedrakyan AM Ktsoyan ZA Arakelova KA Zakharyan MK Hovhannisyan AI Gevorgyan ZU. Extended-spectrum β-lactamases in human isolates of multidrug-resistant non-typhoidal *Salmonella enterica*. Front Microbiol. (2020) 11:592223. 10.3389/fmicb.2020.592223 33414769 PMC7783090

[44] Yosi F Sandi S Sahara E Sari ML Gofar N. Effect of lactic acid bacteria isolated from ensiled kumpai tembaga on growth performance and meat quality of pegagan ducks. Int J Vet Sci. (2022) 11:243–8 10.47278/journal.ijvs/2021.109

[45] Stevens MP Kingsley RA. *Salmonella* pathogenesis and host-adaptation in farmed animals. Curr Opin Microbiol. (2021) 63:52–8. 10.1016/j.mib.2021.05.013 34175673

[46] Holschbach CL Peek SF. *Salmonella* in dairy cattle. Vet Clin Food Anim Pract. (2018) 34:133–54. 10.1016/j.cvfa.2017.10.005 29224803 PMC7135009

[47] Jajere SM. A review of *Salmonella enterica* with particular focus on the pathogenicity and virulence factors, host specificity and antimicrobial resistance including multidrug resistance. Vet World. (2019) 12:504. 10.14202/vetworld.2019.504-521 31190705 PMC6515828

[48] Jepson MA Clark MA. The role of M cells in *Salmonella* infection. Microbes Infect. (2001) 3:1183–90. 10.1016/S1286-4579(01)01478-2 11755406

[49] Zbrun MV Rossler E Romero-Scharpen A Soto LP Berisvil A Zimmermann JA. Worldwide meta-analysis of the prevalence of *Campylobacter* in animal food products. Res Vet Sci. (2020) 132:69–77. 10.1016/j.rvsc.2020.05.017 32521281

[50] Mourkas E Florez-Cuadrado D Pascoe B Calland JK Bayliss SC Mageiros L. Gene pool transmission of multidrug resistance among *Campylobacter* from livestock, sewage and human disease. Environ Microbiol. (2019) 21:4597–613. 10.1111/1462-2920.14760 31385413 PMC6916351

[51] Hakeem MJ Lu X. Survival and control of *Campylobacter* in poultry production environment. Front Cell Infect Microbiol. (2021) 10:615049. 10.3389/fcimb.2020.615049 33585282 PMC7879573

[52] Royden A Christley R Prendiville A Williams NJ. The role of biosecurity in the control of Campylobacter: a qualitative study of the attitudes and perceptions of UK broiler farm workers. Front Vet Sci. (2021) 8:751699. 10.3389/fvets.2021.751699 34993244 PMC8724210

[53] Teh AHT Lee SM Dykes GA. Association of some *Campylobacter jejuni* with Pseudomonas aeruginosa biofilms increases attachment under conditions mimicking those in the environment. PLoS ONE. (2019) 14:e0215275. 10.1371/journal.pone.0215275 30970009 PMC6457560

[54] Chen D McKune SL Singh N Yousuf Hassen J Gebreyes W Manary MJ. *Campylobacter* colonization, environmental enteric dysfunction, stunting, and associated risk factors among young children in rural Ethiopia: a cross-sectional study from the campylobacter genomics and environmental enteric dysfunction (CAGED) project. Front Public Health. (2021) 8:615793. 10.3389/fpubh.2020.615793 33553097 PMC7862945

[55] Elmi A Nasher F Dorrell N Wren B Gundogdu O. Revisiting *Campylobacter jejuni* virulence and fitness factors: role in sensing, adapting, and competing. Front Cell Infect Microbiol. (2021) 10:607704. 10.3389/fcimb.2020.607704 33614526 PMC7887314

[56] Schiaffino F Platts-Mills J Kosek MN. A One Health approach to prevention, treatment, and control of campylobacteriosis. Curr Opin Infect Dis. (2019) 32:453–60. 10.1097/QCO.0000000000000570 31305492

[57] Muthuirulandi Sethuvel DP Devanga Ragupathi NK Anandan S Veeraraghavan B. Update on: *Shigella* new serogroups/serotypes and their antimicrobial resistance. Lett Appl Microbiol. (2017) 64:8–18. 10.1111/lam.12690 27783408

[58] Lee MS Yoon JW Tesh VL. Recent advances in understanding the pathogenesis of shiga toxin-producing *Shigella* and *Escherichia coli*. Front Cell Infect Microbiol. (2020) 10:620703. 10.3389/fcimb.2020.620703 33324585 PMC7726016

[59] Zaib H Kanwar R Zafar N Ali S. Prevalence and multidrug resistance profiles of several bacterial pathogens isolated from hospital inanimate surfaces in Faisalabad, Pakistan. Novel Res Microbiol J. (2019) 3:526–34. 10.21608/nrmj.2019.66745

[60] Yang SC Hung CF Aljuffali IA Fang JY. The roles of the virulence factor IpaB in *Shigella* spp. in the escape from immune cells and invasion of epithelial cells. Microbiol Res. (2015) 181:43–51. 10.1016/j.micres.2015.08.006 26640051

[61] Kayode AJ Semerjian L Osaili T Olapade O Okoh AI. Occurrence of multidrug-resistant *Listeria monocytogenes* in environmental waters: a menace of environmental and public health concern. Front Environ Sci. (2021) 9:737435. 10.3389/fenvs.2021.737435

[62] Ranasinghe RASS Satharasinghe DA Tang JYH Rukayadi Y Radu KR New CY. Persistence of *Listeria monocytogenes* in food commodities: food-borne pathogenesis, virulence factors, and implications for public health. Food Res. (2021) 5:1–16. 10.26656/fr.2017.5(1)0.199

[63] Quereda JJ Morón-García A Palacios-Gorba C Dessaux C García-del Portillo F Pucciarelli MG. Pathogenicity and virulence of *Listeria monocytogenes*: a trip from environmental to medical microbiology. Virulence. (2021) 12:2509–45. 10.1080/21505594.2021.1975526 34612177 PMC8496543

[64] Bell C Kyriakides A. Listeria: A Practical Approach to the Organism and its Control in Foods, 2nd Edition. New York, NY: Wiley-Blackwell (2005).

[65] Gohar S Abbas G Sajid S Sarfraz M Ali S Ashraf M. Prevalence and antimicrobial resistance of *Listeria monocytogenes* isolated from raw milk and dairy products. Matrix Sci Med. (2017) 1:10–4. 10.26480/msm.01.2017.10.14

[66] Chung LK Bliska JB. *Yersinia* versus host immunity: how a pathogen evades or triggers a protective response. Curr Opin Microbiol. (2016) 29:56–62. 10.1016/j.mib.2015.11.001 26638030 PMC4755919

[67] Joutsen S Fredriksson-Ahomaa M. *Yersinia enterocolitica*/ Properties and occurrence. Encyclopedia Food Health. (2016) 5:606–11. 10.1016/B978-0-12-384947-2.00763-7

[68] Karlsson PA Tano E Jernberg C Hickman RA Guy L Järhult JD. Molecular characterization of multidrug-resistant *Yersinia enterocolitica* from food-borne outbreaks in Sweden. Front Microbiol. (2021) 12:664665. 10.3389/fmicb.2021.664665 34054769 PMC8155512

[69] Shoaib M Shehzad A Raza H Niazi S Khan IM Akhtar W. A comprehensive review on the prevalence, pathogenesis, and detection of *Yersinia enterocolitica*. RSC Adv. (2019) 9:41010–21. 10.1039/C9RA06988G 35540058 PMC9076465

[70] Ali S Nawaz Z Akhtar A Aslam R Zahoor MA Ashraf M. Epidemiological investigation of human brucellosis in Pakistan. Jundishapur J Microbiol. (2018) 11:e61764. 10.5812/jjm.6176431847082

[71] Yawoz M Jaafar SE Alali F Babur C. Seroprevalence of camels listeriosis, brucellosis and toxoplasmosis from Kirkuk Province-Iraq. Pak Vet J. (2021) 41:335–40. 10.29261/pakvetj/2021.030

[72] Khan UD Khan A Gul ST Saleemi MK Du XX. Seroprevalence of brucellosis in cattle (*Bos taurus*) kept in peri urban areas of Pakistan. Agrobiol Rec. (2020) 1:6–10. 10.47278/journal.abr/2020.002

[73] Traxler RM Guerra MA Morrow MG Haupt T Morrison J Saah JR. Review of brucellosis cases from laboratory exposures in the United States in 2008 to 2011 and improved strategies for disease prevention. J Clin Microbiol. (2013) 51:3132–6. 10.1128/JCM.00813-13 23824776 PMC3754678

[74] Luelseged A Zeleke E Tessema F Getaneh B Enbiyale G. Review on molecular epidemiology and public health significance of brucellosis. J Anim Res Vet Sci. (2018) 2:2–10. 10.24966/ARVS-3751/100007

[75] Schaeffer J Revilla-Fernández S Hofer E Posch R Stoeger A Leth C. Tracking the origin of Austrian human brucellosis cases using whole genome sequencing. Front Med. (2021) 8:635547. 10.3389/fmed.2021.635547 33718408 PMC7943447

[76] Moreno E. Retrospective and prospective perspectives on zoonotic brucellosis. Front Microbiol. (2014) 5:213. 10.3389/fmicb.2014.00213 24860561 PMC4026726

[77] Zhou K Wu B Pan H Paudyal N Jiang J Zhang L. ONE health approach to address zoonotic brucellosis: a spatiotemporal associations study between animals and humans. Front Vet Sci. (2020) 7:521. 10.3389/fvets.2020.00521 32984409 PMC7492289

[78] Teske SS Huang Y Tamrakar SB Bartrand TA Weir MH Haas CN. Animal and human dose-response models for *Brucella* species. Risk Anal Int Jo. (2011) 31:1576–96. 10.1111/j.1539-6924.2011.01602.x 21449960

[79] Yoo JR Heo ST Lee KH Kim YR Yoo SJ. Food-borne outbreak of human brucellosis caused by ingested raw materials of fetal calf on Jeju Island. Am J Trop Med Hyg. (2015) 92:267. 10.4269/ajtmh.14-0399 25510725 PMC4347327

[80] Khan I Ali S Hussain R Raza A Younus M Khan N. Serosurvey and potential risk factors of brucellosis in dairy cattle in peri-urban production system in Punjab, Pakistan. Pak Vet J. (2021) 10:459–62. 10.29261/pakvetj/2021.028

[81] Jabeen R Yasmin M Dar HR Siddiqui RT Ullah I. Characterization of Mutations linked with second line anti-TB drug resistance in Pakistan. Adv Life Sci. (2021) 8:137–42. Available online at: http://www.als-journal.com/828-21/

[82] Duffy SC Srinivasan S Schilling MA Stuber T Danchuk SN Michael JS. Reconsidering *Mycobacterium bovis* as a proxy for zoonotic tuberculosis: a molecular epidemiological surveillance study. Lancet Microbe. (2020) 1:e66–73. 10.1016/S2666-5247(20)30038-0 32642742 PMC7325494

[83] Chai Q Wang L Liu CH Ge B. New insights into the evasion of host innate immunity by *Mycobacterium tuberculosis*. Cellu Mol Immunol. (2020) 17:901–13. 10.1038/s41423-020-0502-z 32728204 PMC7608469

[84] Rehman AU Ehtisham-ul-Haque S Javed MT Ahmad MZ Ahmed I Rafique MK. (2021). Monitoring the health status and herd-level risk factors of tuberculosis in water buffalo (*Bubalus bubalis*) dairy farms in Pakistan. Pak Vet J. 41, 552–556. 10.29261/pakvetj/2021.051

[85] Macedo Couto R Ranzani OT Waldman EA. Zoonotic tuberculosis in humans: control, surveillance, and the one health approach. Epidemiol Rev. (2019) 41:130–44. 10.1093/epirev/mxz002 32294188

[86] Haque MA Quan H Zuo Z Khan A Siddique N He C. Pathogenicity of feed-borne *Bacillus cereus* and its implication on food safety. Agrobiol Rec. (2021) 3:1–16. 10.47278/journal.abr/2020.015

[87] Khan MUZ Liu B Yang S Xu X Wang Y Cai J. Genetic diversity of *Clostridium perfringens* strains isolated from broiler chickens revealed by PFGE analysis in China and Pakistan. Pak Vet J. (2021) 41:85–91. 10.29261/pakvetj/2020.087

[88] Zell C Resch M Rosenstein R Albrecht T Hertel C Götz F. Characterization of toxin production of coagulase-negative staphylococci isolated from food and starter cultures. Int J Food Microbiol. (2008) 127:246–51. 10.1016/j.ijfoodmicro.2008.07.016 18752861

[89] Chin D Goncheva MI Flannagan RS Deecker SR Guariglia-Oropeza V Ensminger AW. Coagulase-negative staphylococci release a purine analog that inhibits *Staphylococcus aureus* virulence. Nat Commun. (2021) 12:1–12. 10.1038/s41467-021-22175-3 33767207 PMC7994395

[90] Chajecka-Wierzchowska W Zadernowska A Gajewska J. S. epidermidis strains from artisanal cheese made from unpasteurized milk in Poland-Genetic characterization of antimicrobial resistance and virulence determinants. Int J Food Microbiol. (2019) 294:55–9. 10.1016/j.ijfoodmicro.2019.02.004 30771666

[91] Zhao H Xu S Yang H He C Xu X Hu F. Molecular typing and variations in amount of tst gene expression of TSST-1-producing clinical Staphylococcus aureus isolates. Front Microbiol. (2019) 10:1388. 10.3389/fmicb.2019.01388 31275293 PMC6594356

[92] Fisher EL Otto M and Cheung GY. Basis of virulence in enterotoxin-mediated staphylococcal food poisoning. Front Microbiol. (2018) 9:436. 10.3389/fmicb.2018.00436 29662470 PMC5890119

[93] Elmossalamy DA Hamdy MM Aideia HAM Yassien NA Zaki HMBA. Incidence of *Staphylococcus aureus* and its enterotoxins in chicken meat and its products. Int J Vet Sci. (2020) 9:573–7.26807798

[94] Hennekinne JA De Buyser ML Dragacci S. *Staphylococcus aureus* and its food poisoning toxins: characterization and outbreak investigation. FEMS Microbiol Rev. (2012) 36:815–36. 10.1111/j.1574-6976.2011.00311.x 22091892

[95] Fetsch A Johler S. *Staphylococcus aureus* as a foodborne pathogen. Curr Clin Microbiol Rep. (2018) 5:88–96. 10.1007/s40588-018-0094-x

[96] González N Marquès M Nadal M Domingo JL. Meat consumption: which are the current global risks? A review of recent (2010–2020) evidences. Food Res Int. (2020) 137:109341. 10.1016/j.foodres.2020.109341 33233049 PMC7256495

[97] Osterhaus AD Vanlangendonck C Barbeschi M Bruschke CJ Christensen R Daszak P. Make science evolve into a One Health approach to improve health and security: a white paper. One Health Outlook. (2020) 2:1–32. 10.1186/s42522-019-0009-7 32835168 PMC7162674

[98] Gizaw Z. Public health risks related to food safety issues in the food market: a systematic literature review. Environ Health Prev Med. (2019) 24:1–21. 10.1186/s12199-019-0825-5 31785611 PMC6885314

[99] Hernando-Amado S Coque TM Baquero F Martínez JL. Defining and combating antibiotic resistance from One Health and Global Health perspectives. Nat Microbiol. (2019) 4:1432–42. 10.1038/s41564-019-0503-9 31439928

[100] Pettengill JB Beal J Balkey M Allard M Rand H Timme R. Interpretative labor and the bane of nonstandardized metadata in public health surveillance and food safety. Clin Infect Dis. (2021) 73:1537–9. 10.1093/cid/ciab615 34240118

[101] Gargiulo AH Duarte SG Campos GZ Landgraf M Franco BD Pinto UM. Food safety issues related to eating on and eating out. Microorganisms. (2022) 10:2118. 10.3390/microorganisms10112118 36363709 PMC9695559

[102] van Herten J Bovenkerk B Verweij M. One Health as a moral dilemma: towards a socially responsible zoonotic disease control. Zoonoses Public Health. (2019) 66:26–34. 10.1111/zph.12536 30390380 PMC7379490

[103] Riggio GM Wang Q Kniel KE Gibson KE. Microgreens—a review of food safety considerations along the farm to fork continuum. Int J Food Microbiol. (2019) 290:76–85. 10.1016/j.ijfoodmicro.2018.09.027 30308448

